# Development of Hydrogels with the Incorporation of *Raphanus sativus* L. Seed Extract in Sodium Alginate for Wound-Healing Application

**DOI:** 10.3390/gels7030107

**Published:** 2021-08-04

**Authors:** Muhammad Zahid, Maria Lodhi, Ayesha Afzal, Zulfiqar Ahmad Rehan, Muzzamil Mehmood, Talha Javed, Rubab Shabbir, Dorota Siuta, Fayez Althobaiti, Eldessoky S. Dessok

**Affiliations:** 1 Department of Chemistry, University of Agriculture, Faisalabad 38000, Pakistan; zahid595@gmail.com (M.Z.); mk0441021@gmail.com (M.L.); 2 Department of Materials, National Textile University, Faisalabad 37610, Pakistan; ayesha.afzal@ntu.edu.pk (A.A.); muzammil@ntu.edu.pk (M.M.); 3 College of Agriculture, Fujian Agriculture and Forestry University, Fuzhou 350002, China; mtahaj@fafu.edu.cn (T.J.); rubabshabbir28@gmail.com (R.S.); 4 Department of Agronomy, University of Agriculture, Faisalabad 38000, Pakistan; 5 Faculty of Process and Environmental Engineering, Lodz University of Technology, 90-924 Lodz, Poland; dorota.siuta@p.lodz.pl; 6 Department of Biotechnology, College of Science, Taif University, P.O. Box 11099, Taif 21944, Saudi Arabia; faiz@tu.edu.sa; 7 Department of Biology, College of Science, Taif University, P.O. Box 11099, Taif 21944, Saudi Arabia; es.dessoky@tu.edu.sa

**Keywords:** sodium alginate, hydrogel, polymer, wound healing, sustainability

## Abstract

Hydrogels prepared from polymers have been proposed for tissue regeneration and the treatment of bruise wounds. In this research work, we synthesized a *Raphanus sativus* L.-based wound-healing hydrogel with recognized antimicrobial activity for the healing of cutaneous lesions, drawing on its healing potential. A structural analysis was performed by Fourier transform infrared spectroscopy, confirming the interaction between sodium alginate and *Raphanus sativus* L. The surface morphology was studied by scanning electron microscopy. A swelling test showed that the T-1 hydrogel capability of absorption of the solution was superior compared to other synthesized samples. It was evident that the swelling tendency decreased as the *Raphanus sativus* L. seed extract concentration was reduced. In a thermogravimetric analysis, T-1 shows high thermal stability over other prepared hydrogel samples, enjoying a high content of seed extract compared with all samples. The prepared hydrogels were placed on the chick chorioallantoic membrane of fertilized chick eggs, and their healing capability was examined. All seed extracts containing hydrogels showed clear curative performance as compared to the control hydrogel, whereas their healing magnitude lessened as the extract ratio decreased. It was concluded from the results of the current study that the *Raphanus sativus* L. plant has wound-healing characteristics.

## 1. Introduction

The world of science is searching for innovative schemes and practices to devise and formulate materials for the redevelopment and healing of wounds, which will give them advanced medical support to improve bio-accessibility at the wound area [1]. Skin is the largest and most sensitive part of the human body and can easily be harmed by persistent diseases or severe injuries [2]. As stated by the World Health Organization (WHO), patients suffering burns have poor access to appropriate treatment, resulting in approximately 265,000 deaths annually [3]. For this reason, wound bandages are utilized to shield damage and skin injuries that have happened after accidents, ailments, and surgery [4]. Wound healing is an effective mechanism whereby missing and impaired tissues and cellular structures are substituted. This is attained in four specific phases, hemostasis, inflammation, propagation, and remodeling, that maintain the functional integrity of the injured muscles [5].

For designing a good wound-healing system, some key factors should be considered. For example, it should be biocompatible, provide a moist environment to the wound, maintain good absorption capacity of wound exudate and fluids, and allow the finest gas diffusion, thus stimulating healing and preventing the intrusion of germs at the wound bed. In all the above factors, biocompatibility plays a crucial role [6]. Abandonment of the above aspects leads to unending wounds. The usage of innovative wound bandages such as foams, hydro-fibers, hydrogels, and alginates is expanding day by day [7]. The three-dimensional structure of hydrogels holds and absorbs a large quantity of water because the different hydrophilic functional groups are assembled along the backbone of the polymer. Therefore, they are extensively utilized in the pharmaceutical field involving wound bandages, drug delivery, and contact lens applications [8].

The major properties according to which the hydrogels are synthesized and selected for wound bandages are high water absorption aptitude, access of moisture to the wound bed, and prevention of dryness in the wound [9]. Hydrogels have a resemblance to the extracellular matrix (ECM), which facilitates the non-toxic and non-allergic factors in them [10]. Hydrogels can be synthesized through chemical approaches, via radiation (electron and gamma rays) and chemical crosslinking techniques. Another method is the physical approach involving the freeze-thaw cycle [11]. Freeze-thaw is the most suitable technique for biomedical applications because it does not involve heating reagents, chemicals, or toxic cross-linkers, which otherwise may create toxicity and side effects [12].

Numerous biopolymers are utilized in the formation of wound bandages. In hydrogel-centered bandages, biopolymers such as sodium alginates are consumed because they are biocompatible, are of comparatively low cost, lack toxic factors derivative of brown algae, and are also present in some species of bacteria [13]. Sodium alginate is attractive in wound healing because of its structural resemblance to extracellular patterns of living organs, maintenance of low temperature, and ideal humid atmosphere [14]. It also maintains humid surroundings that enhance reepithelization and fast granulation, lessen the risk of bacterial infection, and assist in wound recovery [15]. The most significant property of sodium alginate from the biomedical viewpoint is the potential of crosslinking even in minor conditions via a cation series of divalent nature. The hydrogel prepared from sodium alginate is inherently hydrophilic and biocompatible in nature [16].

Plants utilized for traditional medicine consist of a broad series of elements that can be beneficial to cure infectious or chronic diseases. *Raphanus sativus* L. (Radish) is a medicinal vegetable of the Brassicaceae family. It is rich in biologically active compounds [17], with anticancer, antimicrobial [18,19], antioxidant, and anti-inflammatory medicinal properties. Traditional Persian medicine utilized the *Raphanus sativus* L. plant for wound healing because of its antioxidant, antimicrobial, anti-inflammatory, and radical scavenging activities [20]. However, modern research has not examined it for its wound-healing activities. That is why in this article, we studied the seed extract of *Raphanus sativus* L. to examine its healing activity in combination with sodium alginate [21]. 

The integration of bioactive fractions into substances of a polysaccharide nature and their efficiency as wound bandages have been measured many times in the literature [22]. In the present work, the seed extract of *Raphanus sativus* L. is incorporated with sodium alginate for making hydrogel [23,24,25]. The main focus of using both materials was to synthesize porous scaffolds so as to measure their activity in wound healing. In this study, we report the formulation of hydrogels incorporating the seed extract of *Raphanus sativus* L. in sodium alginate using the freeze-thaw cycle and investigate their surface, shape, chemical interaction, water absorption capacity, heating stability, as well as their healing activity using a chorioallantoic membrane assay (CAM). Four hydrogels were synthesized using different ratios of extract and polymer solution (3%) dissolved in water. A control hydrogel (without extract) was also defined to compare properties. A CAM assay was performed on sterilized eggs under controlled conditions. 

## 2. Results and Discussion

### 2.1. Fourier Transform Infrared Spectroscopy 

**Figure 1:**
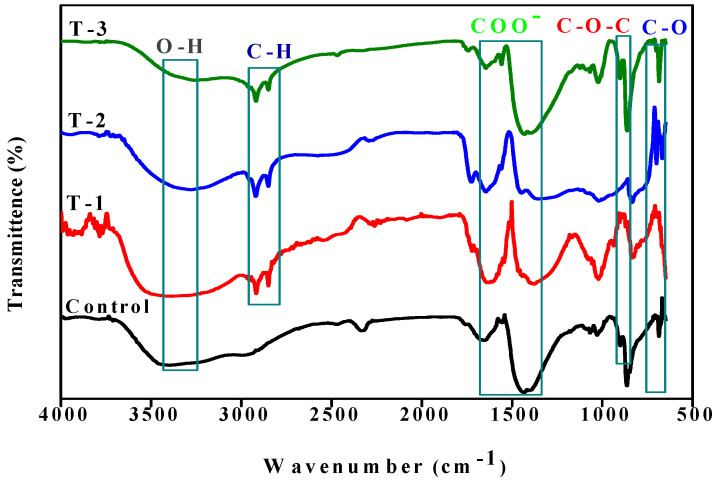
FTIR analysis of control; T-1: (SA:SE 70:30); T-2: (SA:SE 80:20); T-3: (SA:SE 90:10) hydrogels.

The chemical interaction between the polymeric material and plant seed extract was studied by a Fourier transform infrared spectroscopy (FTIR) assay. Control samples show characteristic peaks of SA hydrogel, as shown in Figure 1, and the broad band at 3411 cm^−1^ is assigned to O–H stretching. The peaks at 1436 cm^−1^ and 1663 cm^−1^ are due to the symmetric and asymmetric stretching vibration of COO (carboxylic group), respectively. The minor peak at 1036 cm^−1^ is the result of C–O–C asymmetric stretching due to its saccharide structure [26]. The intense peak at 861 cm^−1^ is assigned to the C–O stretching vibration appeared due to the saccharide structure presence in the carbohydrate backbone [27]. In the literature, the characteristic peaks of extract reviewed for O–H stretching in a range of 3315–3385 cm^−1^ due to the presence of phenolic compound and peaks in the region of 2850 to 2930 cm^−1^ are assigned to C–H stretching modes of methyl or methoxy group present in *Raphanus sativus* L. [28]. 

Figure 1 shows that the peaks position fall for all the samples T-1, T-2, and T-3 almost in the same range, and the same has been observed in the literature [29], showing no significant change with the addition of different amounts of SA and SE. In the T-1, T-2, and T-3 hydrogels, a broader O–H stretching peak appeared in the region of 3250–3390 cm^−1^ as compared to the pure SA hydrogel, and a shift occurred in the position due to the interaction of polymeric material with the extract. The peaks at 2850 and 2927 cm^−1^ that are absent in the control hydrogel arise due to the C–H stretching modes of methoxy or methyl groups respectively and show the presence of SE in the SA-SE hydrogels sample. The peaks that appeared at 1450 cm^−1^ and 1643 cm^−1^ were due to the symmetric and asymmetric stretching vibration of COO- respectively, and the shifting that occurred in their position is dependent upon the M/G monomer unit [29]. The peaks observed at 1023 cm^−1^ and 836 cm^−1^ are assigned to C–O–C asymmetric stretching and C–O stretching vibration, respectively, which were less intense in the T-1 and T-2 hydrogels as compared to the control samples, and this shift has been observed in the intensity of bands due to the SA crosslinking with SE of the plant [30]. Thus, the FTIR spectra of SA-SE hydrogels indicated the interaction between sodium alginate and *Raphanus sativus* L. seed extract.

### 2.2. Morphological Analysis

**Figure 2:**
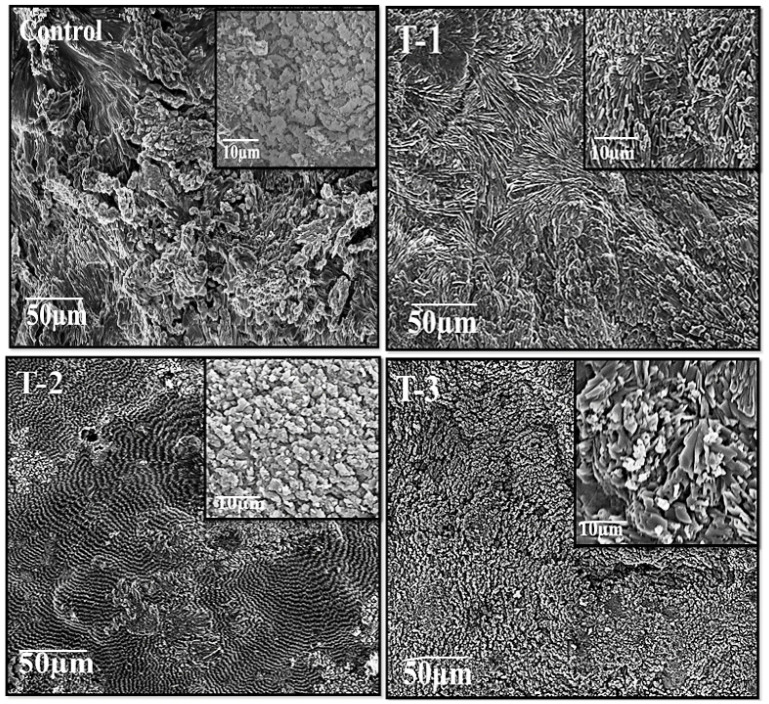
Scanning electron microscope (SEM) images of Control; T-1: (SA:SE 70:30); T-2: (SA:SE 80:20); and T-3: (SA:SE 90:10) hydrogels at × 1000 and × 2500 magnifications.

Morphological structures of SA-SE hydrogels were studied using SEM micrographs. Figure 2 showed SEM images of the cross-sectional morphology and pore structure of the hydrogels. A control hydrogel containing only SA polymer showed crystal formation due to the freeze-thaw cycle. There is a porous surface in the control hydrogel. Hydrogels with varying concentrations of both SA and SE with ratios such as in T-1 (70/30), T-2 (80/20), and T-3 (90/10) exhibited a difference in their morphology that can be seen in the images in Figure 2. The T-1(70/30) hydrogel with a high concentration of SE shows fewer pores on the surface, and most of the surface is occupied by the SE that turns the surface smooth as compared to the control hydrogel sample. However, the number of pores in T-1 is greater as compared to T-2 and T-3. Samples T-2 and T-3 show comparatively different surfaces. Both T-2 and T-3 show a compact and smoother surface than the porous surface of the T-1 hydrogel. This may be due to the decreasing ratio of SE in T-2 and T-3. It is evident from the SEM that as the SE ratio decreases in the hydrogel samples, their porosity also decreases in the descending order of SE weight percentage ratio. Moreover, the surface appeared deformed, which may be due to the surface drying while drying in oven, which caused more loss of water [31]. 

### 2.3. Swelling Studies

**Figure 3:**
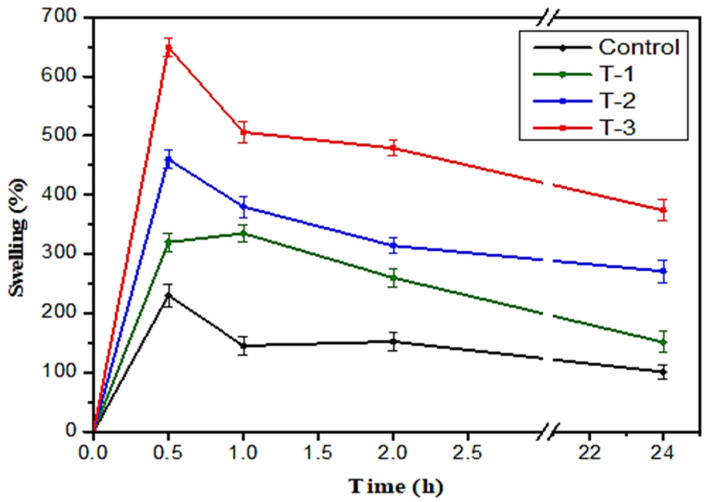
The swelling trend of sodium alginate (SA)-*Raphanus sativus* L. seed extract (SE) hydrogels at 0.5, 1, 2, 3, 12, and 24 h in NaCl solution. Control; T-1: (SA:SE 70:30); T-2: (SA:SE 80:20); and T-3: (SA:SE 90:10).

A swelling test was performed to determine the water absorption capacity of the hydrogels. Control hydrogels T-1, T-2, and T-3 were weighed and then soaked individually in NaCl saline solution for 24 h. Swelling behavior was determined at different time intervals of 0.5, 1, 2, and 24 h. After each specified time interval, their swelling percentages were noted by removing each sample from the solution with the help of forceps and measuring their wet weight using an electronic balance. All samples revealed a time-dependent swelling profile, attaining their highest absorption value within half an hour to 1 h (Figure 3). For the most part, samples exhibited the above-stated pattern. 

Figure 3 displays the swelling trend of the hydrogels. A control hydrogel based on only SA and T-1, T-2, and T-3 contain varying concentrations of both SA and SE solution in different weight percentage ratios such as 70/30, 80/20, and 90/10, respectively. The control sample reached its maximum swelling value of 245% at 0.5 h (Figure 3). This shows the lowest water absorption in the control sample as compared to the T-1, T-2, and T-3 hydrogel samples. Sample T-1 shows a maximum absorption value of 660% at 0.5 h and a decrease of 212%, 213%, and 176% swelling at time intervals of 1, 2, and 24 h, respectively. Sample T-2 shows the swelling ratio value of 474% at 0.5 h, and after that, the swelling value decreases to 244%, 214%, and 197% at a time interval of 1, 2, and 24 h, respectively. Sample T-3 shows a maximum absorption value of 352% after 1 h, and a decrease of 66%, 61%, and 42% can be seen after time intervals of 0.5, 2, and 24 h, respectively (Figure 3). From the above trends, it is clear the control sample shows the lowest water absorption capacity and T-1 shows the highest water absorption capacity in all the samples. It can be concluded that the addition of SE increases the swelling capability of the samples. As the quantity of SE decreases, the water swelling capability also decreases in descending order of SE weight percentage ratio in the samples (Figure 3).

### 2.4. Thermogravimetric Analysis (TGA)

**Figure 4:**
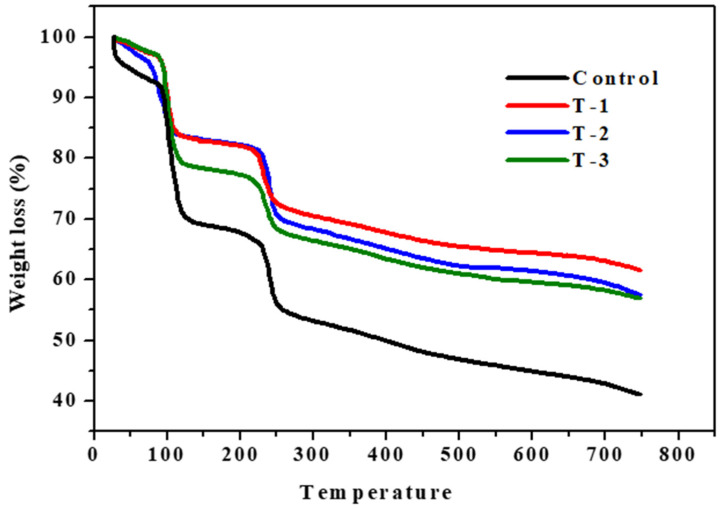
Thermogravimetric analysis (TGA) of T-1: (SA:SE 70:30); T-2: (SA:SE 80:20); and T-3: (SA:SE 90:10) hydrogels in comparison to control hydrogel.

The thermal degradation behavior of the control, T-1, T-2, and T-3 hydrogels were examined by means of TGA curves to examine decomposition trends with varying SE concentrations at different temperatures, as shown in Figure 4. The thermal degradation profile of all the hydrogels showed three steps of degradation, as shown in Figure 4. Polysaccharide decomposition usually follows the removal of structural and physically absorbed H_2_O (dehydration), and the breakdown of C–O and C–C bonds (depolymerization) present in the ring structure leads to the formation of water, CO_2_, and CO [26]. The first weight loss of all hydrogel samples due to the loss of water takes place in a temperature range of 75 to 115 °C. The second decomposition stage as shown in Figure 4 occurs between a temperature range of 120 and 220 °C, which shows a 3% weight loss due to the evaporation of absorbed water and start of the breakdown of SA polymers.

The third stage of thermal degradation occurs in a temperature range of 225 to 250 °C. The weight loss that occurs in the range of 200 to 300 °C in hydrogels is due to the breakdown of the sodium alginate backbone and side chain present in the ring structure [32]. From 300 to 750 °C, a gradual weight loss is seen. This is due to the decarboxylation and formation of carbon dioxide [33].

When the weight loss of the control hydrogel is compared to the T-1, T-2, and T-3 hydrogels, it can be seen in Figure 4 that the T-1 hydrogel shows the highest thermal stability, and the control hydrogel shows the lowest thermal stability among all hydrogel samples. The thermal stability decreases as the SE ratio decreases in the hydrogel samples, T-2 shows less thermal stability than T-1, and T-3 shows less thermal stability than T-2, as shown in Figure 4. The temperature ranges of all hydrogel samples and their percentage of weight loss are given in Table 1. From the results in the graph, it is clear that the content of SE in hydrogel imparts stability, and this enhancement in their thermal stability is due to the strong interaction between SA and SE in the blend. These significant interactions may protect them from decomposition while heating and reduce their mass loss during thermogravimetric analysis. From the results in Figure 4, it can be concluded that where the SE ratio is lower in the samples, their thermal stability is also lower and thermal stability decreases in the descending order of the SE weight percentage ratio. The starting regions (T-_onset_) and the end regions (T-_endset_) of thermal decomposition for all the hydrogels are given in Table 1. W represents the percentage of weight loss.

### 2.5. Chorioallantoic Membrane (CAM) Assay 

**Figure 5:**
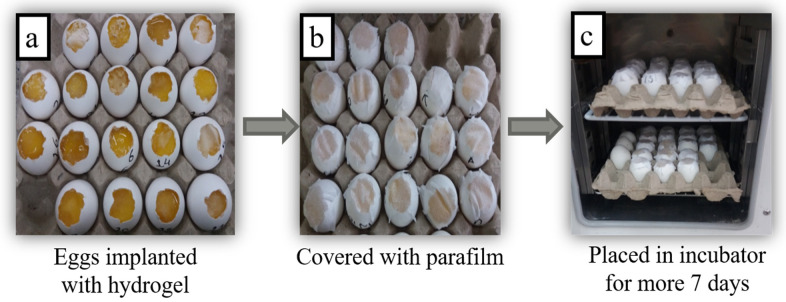
Stepwise handling of chick eggs at day 7.

A CAM assay was executed to measure the capability of hydrogels incorporating SE to check their healing potential. Figure 5a shows the microscopic images taken on the 7th day when hydrogels were implanted on fertilized chick eggs after keeping them for 7 days in an incubator. After implanting hydrogels on the cutting surface of eggs, they were covered with paraffin and paper tape to prevent any fungal infection, as can be seen in Figure 5b. After that, all eggs with implanted hydrogels were placed in an incubator at 37 °C for seven more days, as Figure 5c shows. After the completion of 14 days, eggs were removed from the incubator, their images were taken by a light microscope, and the healing response of the implanted hydrogels was examined on each egg. As stated above, we prepared hydrogels from the SE and SA collectively in different weight percentage ratios. 

**Figure 6:**
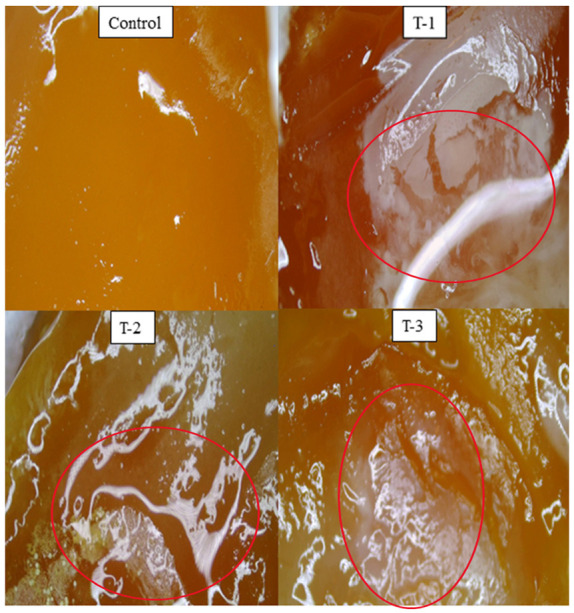
Microscopic images of fertilized eggs implanted with sodium alginate (SA)-*Raphanus sativus* L. seed extract (SE) hydrogels on day 14. Control, T-1: (SA:SE 70:30); T-2: (SA:SE 80:20); T-3: (SA:SE 90:10).

Microscopic images of eggs implanted with control hydrogel do not show any healing, as shown in Figure 6. The T-1, T-2, and T-3 hydrogels implanted on eggs with different weight percentage ratios of SA and SE show some healing tendency that can be seen in microscopic images taken from the light microscope on day 14. It can also be observed in Figure 6 that sample T-1 (70/30 ratio of SA/SE) shows more healing as compared to the T-2 (80/20 ratio of SA/SE) and T-3 (90/10 ratio of SA/SE) samples. CAM assay indicated that T-3 hydrogel not only enticed a significant amount of blood vessels but also boosted the growth of new ones. A dense network of blood vessels was developed in and around the hydrogel Figure 6. In addition, T-1 demonstrated an attachment of scaffold with the CAM, but the number of blood vessels involved toward the scaffold was less than that of T-3. It may be because of the elevated concentration of seed extract. The control scaffold was neither attached to the CAM nor validated the development of new blood vessels. A similar study was described elsewhere [24,25]. Furthermore, it is an in vivo model of experimentation and is considered as an appropriate substitute to the conventional rodent experimentation, which requires complex experimental conditions and a longer time frame to achieve the results. This is a low cost, quick, and simple technique and meets the criteria of the 3R principle (reduce, refine, replace) [1]. All SEs containing hydrogels showed some extent of healing, but healing decreases as the SE ratio decreases. Thus, we can say that SE has healing potential and can be utilized for wound healing applications. As stated in traditional Persian medicine, *Raphanus sativus* L. has been utilized for anti-inflammatory and antimicrobial activities but has not been utilized for wound-healing activity [20]. For this purpose, we chose this plant extract to check its healing potential, and our results showed positive outcomes. It is also stated that *Raphanus sativus* L. is abundant in secondary metabolites such as alkaloids, glycosides, tannins, and flavonoids [1]. In other plants, these compounds are responsible for healing, so we can say that these compounds may be responsible for the healing capability of radish plants. Furthermore, the transcriptional regulatory molecular mechanism behind the overall improvement of wound-healing attributes is also important from sustainability perspectives [34].

## 3. Materials and Methods

### 3.1. Materials and Chemicals

Sodium alginate was purchased from DAEJUNG Korea. The seeds of a *Raphanus sativus* L. plant were purchased from a local store. Absolute alcohol was obtained from Sigma Aldrich with a purity level of 99.9%. Sodium hydroxide (NaOH) and acetic acid (CH_3_COOH) and NaCl were obtained from Sigma Aldrich (St. Louis, MI, USA). Water from a Millipore Milli^®^-Q system (Merck KGaA, Darmstadt, Germany) was utilized for the synthesis of the gels and solutions.

### 3.2. Preparation Methods

#### 3.2.1. *Raphanus sativus* L. Extract Preparation

First of all, *Raphanus sativus* L. (radish) plant seeds were washed with distilled water and dried. The 10 g seeds were crushed in a pestle and mortar. The crushed seeds were heated in 100 mL of distilled water, and the solution was stirred continuously for 6 h at 60 °C with a magnetic stirrer. After 6 h of heating, the solution was left to cool down at room temperature. The solution was filtered using Whatman filter paper. The filtrate was collected in a bottle and placed in a refrigerator at 4 °C [23].

#### 3.2.2. Sodium Alginate Solution Preparation

The stock solution containing 3 g of sodium alginate in 100 mL of distilled water was prepared. The solution was placed on a magnetic stirrer for five hours at room temperature for the complete mixing of sodium alginate. After 5 h, a transparent homogeneous mixture of sodium alginate was obtained. Then, the solution was stored in the refrigerator at 4 °C.

### 3.3. Synthesis of SA-SE (Sodium Alginate-Seed Extract) Hydrogel by Freeze-Thawing

The smart hydrogel was synthesized by mixing already-prepared SA solution and SE by a freeze-thaw method. The below-mentioned concentrations in Table 2 show the weight percentage ratio of both SE and SA solutions.

**Figure 7:**
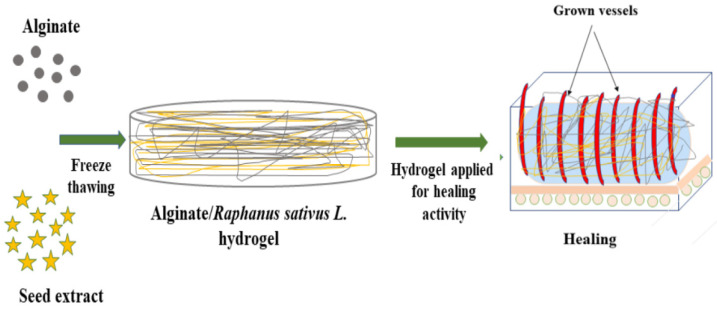
Schematic representation of SA-SE hydrogels.

The concentrations mentioned in Table 2 were mixed in Petri dishes and stirred. All samples were stored at −20 °C overnight. Then, frozen samples were coagulated with NaOH solution and were frozen again at −20 °C for 12 h. A three molar NaOH solution was prepared in distilled water. Then, samples were washed with absolute alcohol two times and next washed with distilled water to maintain their pH neutral. In the end, the washed samples were thawed at 37 °C in the oven for 24 h. The schematic illustration of the SA-SE hydrogels is shown in Figure 7.

### 3.4. Characterization of Synthesized Hydrogels

Fourier transform infrared spectroscopy (FTIR, Perkin Elmer Spectrum two, USA) was carried out for the study of the chemical structure and functional groups on the material’s surface. The analysis was conducted within a range of 650–4000 cm^−1^.

Scanning Electron Microscopy (JSM-5910, JEOL, Japan) was performed to analyze the surface morphology and description of the shape of the synthesized hydrogels. Samples were subjected to 10 k volts.

Swelling measurements of prepared hydrogels were performed in brine solution at 37 °C The three-molar solution of sodium chloride (NaCl) was prepared in distilled water. During the studies, the difference in the weight of dry (*Wd*) and wet (*W*) hydrogels was examined after intervals of 0.5 h, 1 h, 2 h, 3 h, and 24 h, respectively [25].

The equation below was used to measure the percentage of age swelling.

$$Swelling\;\left(\%\right)=\frac{W-W_{d}}{W_{d}}\times 100$$

Thermogravimetric analysis (TGA) (NETZSCH TG 209F1 Libra) was used to determine the thermal stability of the SA-SE hydrogels. Samples were heated at 25 to 350 °C under a nitrogen flow of 20 mL/min at a heating rate of 10 °C/min. The mass of the pan of samples was noted down as a temperature function continuously. 

A CAM assay was carried out to determine the healing activity of the prepared hydrogels. At day zero, chick eggs were purchased from a local bakery, carefully cleaned with absolute ethanol, and then placed in an incubator at 37 °C in a humid environment. After seven days, on day 8, sterilized hydrogels were implanted on the eggs by cutting down a small piece of the eggshell of each egg with a surgical blade. After that, the eggs were sealed by sterilized parafilm, covered with adhesive tape to inhibit any infection, and placed in an incubator at pre-set conditions. The eggs were reopened on day 14, and their healing response pictures were captured with a light microscope to examine those changes that occurred by implanting hydrogels [25].

## 4. Conclusions and Future Prospects

This research was conducted for the synthesis of safe, sustainable, and biocompatible hydrogel incorporated with seed extract of *Raphanus sativus* L. and sodium alginate for easy application in wound healing. Hydrogen characterization was performed to analyze its properties. Synthesized hydrogels comprising SE show a good swelling tendency compared to control. SEM results demonstrate that higher ratios of SE create pores in hydrogels, and their porosity decreases in descending order of SE concentration. The T-1 hydrogel displayed high thermal stability in all samples. CAM assay results concluded that radish extract containing hydrogels in combination with sodium alginate exhibited good healing properties. For future aspects, these wound dressings can be utilized in wound healing applications.

## Figures and Tables

**Table 1:** TGA analysis of Control, T-1, T-2, and T-3 hydrogels.

Sample	Stages	T-_onset_ (°C)	T-_endset_ (°C)	W (%)
Control	I	92.2	119.2	20
	II	137.3	217.3	3
	III	229.8	249.8	9
T-1	I	93.7	112.3	9
	II	122.3	214.8	2
	III	221.8	242.3	7
T-2	I	72.2	107.4	11
	II	117.3	222.3	2
	III	229.8	249.8	10
T-3	I	92.2	117.3	16
	II	124.3	212.3	3
	III	219.8	247.3	8

**Table 2:** Sample concentration of sodium alginate (SA) and *Raphanus sativus* L. seed extract (SE).

Samples	SA (wt %)	SE (wt %)
Control	100	nil
T-1	70	30
T-2	80	20
T-3	90	10

## Data Availability

Not applicable.

## Abbreviations

SA	Sodium alginate

## References

[1] Zahid M., Lodhi M., Rehan Z.A., Tayyab H., Javed T., Shabbir R., Mukhtar A., El Sabagh A., Adamski R., Sakran M.I. (2021). Sustainable development of chitosan/calotropis procera-based hydrogels to stimulate formation of granulation tissue and angiogenesis in wound healing applications. Molecules.

[2] Khan M.I.H., Islam J.M., Kabir W., Rahman A., Mizan M., Rahman M.F., Amin J., Khan M.A. (2016). Development of hydrocolloid Bi-layer dressing with bio-adhesive and non-adhesive properties. Mater. Sci. Eng. C.

[3] Das U., Behera S.S., Singh S., Rizvi S.I., Singh A.K. (2016). Progress in the development and applicability of potential medicinal plant extract-conjugated polymeric constructs for wound healing and tissue regeneration. Phytother. Res..

[4] Zhang D., Zhou W., Wei B., Wang X., Tang R., Nie J., Wang J. (2015). Carboxyl-modified poly (vinyl alcohol)-crosslinked chitosan hydrogel films for potential wound dressing. Carbohydr. Polym..

[5] Shah S.A., Sohail M., Khan S., Minhas M.U., De Matas M., Sikstone V., Hussain Z., Abbasi M., Kousar M. (2019). Biopolymer-based biomaterials for accelerated diabetic wound healing: A critical review. Int. J. Biol. Macromol..

[6] Ambekar R.S., Kandasubramanian B. (2019). Advancements in nanofibers for wound dressing: A review. Eur. Polym. J..

[7] Iacob A.-T., Drăgan M., Ionescu O.-M., Profire L., Ficai A., Andronescu E., Confederat L.G., Lupașcu D. (2020). An overview of biopolymeric electrospun nanofibers based on polysaccharides for wound healing management. Pharmaceutics.

[8] Niknia N., Kadkhodaee R. (2017). Factors affecting microstructure, physicochemical and textural properties of a novel Gum tragacanth-PVA blend cryogel. Carbohydr. Polym..

[9] Jahani-Javanmardi A., Sirousazar M., Shaabani Y., Kheiri F. (2016). Egg white/poly (vinyl alcohol)/MMT nanocomposite hydrogels for wound dressing. J. Biomater. Sci. Polym Ed..

[10] Chaturvedi A., Bajpai A.K., Bajpai J., Singh S.K. (2016). Evaluation of poly (vinyl alcohol) based cryogel–zinc oxide nanocomposites for possible applications as wound dressing materials. Mater. Sci. Eng. C.

[11] Kim J.O., Park J.K., Kim J.H., Jin S.G., Yong C.S., Li D.X., Choi J.Y., Woo J.S., Yoo B.K., Lyoo W.S. (2008). Development of polyvinyl alcohol–sodium alginate gel-matrix-based wound dressing system containing nitrofurazone. Int. J. Pharm..

[12] Cleetus C.M., Primo F.A., Fregoso G., Raveendran N.L., Noveron J.C., Spencer C.T., Ramana C.V., Joddar B. (2020). Alginate hydrogels with embedded ZnO nanoparticles for wound healing therapy. Int. J. Nanomed..

[13] Dantas M.D.M., Cavalcante D.R.R., Araújo F.E.N., Barretto S.R., Aciole G.T.S., Pinheiro A.L.B., Ribeiro M.A.G., Lima-Verde I.B., Melo C.M., Cardoso J.C. (2011). Improvement of dermal burn healing by combining sodium alginate/chitosan-based films and low level laser therapy. J. Photochem. Photobiol. B. Biol..

[14] Qin Y. (2008). Alginate fibres: An overview of the production processes and applications in wound management. Polym. Int..

[15] Donati I., Paoletti S. (2009). Material properties of alginates. Alginates: Biology and applications.

[16] Beevi S.S., Mangamoori L.N., Dhand V., Ramakrishna D.S. (2009). Isothiocyanate profile and selective antibacterial activity of root, stem, and leaf extracts derived from *Raphanus sativus* L. Foodborne Pathog. Dis..

[17] Abdou I.A., Abou-Zeid A.A., El-Sherbeeny M.R., Abou-El-Gheat Z.H. (1972). Antimicrobial activities of *Allium sativum*, *Allium cepa*, *Raphanus sativus*, *Capsicum frutescens*, *Eruca sativa*, *Allium kurrat* on bacteria. Qual. Plant. Mater. Veg..

[18] Ghazanfar S.A., Al-Al-Sabahi A.M. (1993). Medicinal plants of northern and central Oman (Arabia). Econ. Bot..

[19] Hosseinkhani A., Falahatzadeh M., Raoofi E., Zarshenas M.M. (2017). An evidence-based review on wound healing herbal remedies from reports of traditional Persian medicine. J. Evid-Based Complementary Altern. Med..

[20] Diniz F.R., Maia R.C.A., Rannier L., Andrade L.N., Chaud M.V., da Silva C.F., Corrêa C.B., de Albuquerque Junior R.L.C., da Costa L.P., Shin S.R. (2020). Silver nanoparticles-composing alginate/gelatine hydrogel improves wound healing in vivo. Nanomater..

[21] Liakos I., Rizzello L., Hajiali H., Brunetti V., Carzino R., Pompa P.P., Athanassiou A., Mele E. (2015). Fibrous wound dressings encapsulating essential oils as natural antimicrobial agents. J. Mater. Chem. B..

[22] Gombotz W.R., Wee S.F. (2012). Protein release from alginate matrices. Adv. Drug Deliv. Rev..

[23] Rani I., Akhund S., Abro H. (2008). Antimicrobial potential of seed extract of *Raphanus sativus*. Pak. J. Bot..

[24] Aleem A.R., Shahzadi L., Alvi F., Khan A.F., Chaudhry A.A., ur Rehman I., Yar M. (2017). Thyroxin releasing chitosan/collagen based smart hydrogels to stimulate neovascularization. Mater. Des..

[25] Javed T., Afzal I., Mauro R.P. (2021). Seed coating in direct seeded rice: An innovative and sustainable approach to enhance grain yield and weed management under submerged conditions. Sustainability.

[26] Tang Q., Pan D., Sun Y., Cao J., Guo Y. (2017). Preparation, characterization and antimicrobial activity of sodium alginate nanobiocomposite films incorporated with ε-Polylysine and cellulose nanocrystals. J. Food Process. Preserv..

[27] Ramya E., Jyothi L., Rao D.N. (2017). Influence of optical properties of Ag NPs from *Raphanus sativus* leaf extract on lanthanide complexes. Plasmonics.

[28] Lawrie G., Keen I., Drew B., Chandler-Temple A., Rintoul L., Fredericks P., Grøndahl L. (2007). Interactions between alginate and chitosan biopolymers characterized using FTIR and XPS. Biomacromolecules.

[29] Sakugawa K., Ikeda A., Takemura A., Ono H. (2004). Simplified method for estimation of composition of alginates by FTIR. J. App. Polym. Sci..

[30] Malagurski I., Levic S., Pantic M., Matijasevic D., Mitric M., Pavlovic V., Dimitrijevic-Brankovic S. (2017). Synthesis and antimicrobial properties of Zn-mineralized alginate nanocomposites. Carbohydr. Polym..

[31] Konwar A., Gogoi A., Chowdhury D. (2015). Magnetic alginate–Fe_3_O_4_ hydrogel fiber capable of ciprofloxacin hydrochloride adsorption/separation in aqueous solution. RSC Adv..

[32] Kusuktham B., Prasertgul J., Srinun P. (2014). Morphology and property of calcium silicate encapsulated with alginate beads. Silicon.

[33] Khan N., Waheed A., Hamid F.S., Ahmed N., Iqbal Z., Ali S., Gul H. (2018). Phytochemical screening and antibacterial assay of radish seed oil on selected pathogenic bacteria species in vitro. Pak. J. Agric. Res..

[34] Javed T., Shabbir R., Ali A., Afzal I., Zaheer U., Gao S.J. (2020). Transcription factors in plant stress responses: Challenges and potential for sugarcane improvement. Plants.

